# Quality appraisal of clinical guidelines for recurrent urinary tract infections using AGREE II: a systematic review

**DOI:** 10.1007/s00192-022-05089-6

**Published:** 2022-02-10

**Authors:** Jorik J. Pat, Lambertus P. W. Witte, Martijn G. Steffens, Robin W. M. Vernooij, Tom A. T. Marcelissen, Paulina Fuentes, Herney A. Garcia-Perdomo, Hector Pardo-Hernandez, Marco H. Blanker

**Affiliations:** 1 Department of General Practice and Elderly Care Medicine, University Medical Centre Groningen, Groningen, The Netherlands; 2 Department of Urology, Isala clinics, Dokter van Heesweg 2, 8025 AB Zwolle, The Netherlands; 3 Department of Nephrology and Hypertension, University Medical Center Utrecht, Utrecht, the Netherlands; 4 Julius Center for Health Sciences and Primary Care, University Medical Center Utrecht, Utrecht University, Utrecht, the Netherlands; 5 Department of Urology, Maastricht University Medical Centre+, Maastricht, The Netherlands; 6 Faculty of Medicine and Dentistry, Universidad de Antofagasta, Antofagasta, Chile; 7 Servicio de Salud Antofagasta, Antofagasta, Chile; 8 Division of Urology Department of Surgery. School of Medicine, Universidad del Valle, Cali, Colombia; 9 Iberoamerican Cochrane Centre, Sant Pau Biomedical Research Institute (IIB Sant Pau), CIBER de Epidemiología y Salud Pública (CIBERESP), Barcelona, Spain

**Keywords:** AGREE, Guideline, Recurrent urinary tract infections, Review

## Abstract

**Introduction and hypothesis:**

Recommendations for preventing and diagnosing recurrent urinary tract infection (UTI) tend to vary between clinical practice guidelines (CPGs) because of low-quality scientific evidence, potentially leading to practice variation and suboptimal care. We assessed the quality of existing CPGs for recurrent UTI.

**Methods:**

A systematic search was performed from January 2000 to June 2021 in PubMed and EMBASE for CPGs on recurrent UTI prevention or hospital diagnostics in Dutch, English, and Spanish. Each CPG was assessed by four appraisers in a multidisciplinary review team, using the Appraisal of Guidelines, Research, and Evaluation II (AGREE II) instrument.

**Results:**

We identified and assessed eight CPGs published between 2013 and 2021. The scope and purpose (mean and standard deviation: 67.3 ± 21.8) and clarity of presentation (74.8 ± 17.6) domains scored highly. However, issues with methods, patient participation, conflict of interests, and facilitators and barriers were common and resulted in lower scores for the rigour of development (56.9 ± 25.9), applicability (19.6 ± 23.4), stakeholder involvement (50.4 ± 24.6), and editorial independence (62.1 ± 23.1) domains. Overall, two CPGs were recommended, three were recommended with modifications, and three were not recommended.

**Conclusions:**

Significant room for improvement exists in the quality of CPGs for recurrent UTI, with most displaying serious limitations in the stakeholder involvement, rigour of development, and applicability domains. These aspects must be improved to decrease diagnostic and therapeutic uncertainty. Developers could benefit from using checklists and following guidelines when developing de novo CPGs.

**Supplementary Information:**

The online version contains supplementary material available at 10.1007/s00192-022-05089-6.

## Introduction

Urinary tract infections (UTIs) are among the most common bacterial infections worldwide, being experienced by one in three women at least once in their lifetime [1]. Recurrent UTI is defined as more than three infections per year or more than two infections per 6 months, and it has a significant impact on quality of life, as highlighted by an international web-based survey of 1941 affected women [2]. There is also a significant economic burden due to the costs of preventive strategies and sick leave [2, 3]. Most recurrent UTIs occur in otherwise healthy women who have no structural genitourinary tract abnormalities [4].

CPGs are defined as “systematically developed statements to assist practitioner and patient decisions about appropriate healthcare in specific clinical circumstances” [5] and are developed to optimize and standardize care. The successful implementation of a CPG depends on rigorous development, a clear implementation strategy, and adequate dissemination. Multiple studies across different clinical areas have shown that variability exists in the quality of CPGs [6–10], indicating that there is considerable room for improvement, especially in rigour of development and applicability. This is important because diagnostic and therapeutic uncertainty can ensue if CPGs differ in core recommendations because of these limitations.

Differences in clinical practice guideline (CPG) recommendations for the preventive treatment of recurrent UTI have been mentioned previously, but the authors of that report did not assess methodological quality [11]. As a result, diagnostic and therapeutic uncertainty might ensue. We could find not a systematic appraisal of the quality of CPGs for the prevention and diagnostics of recurrent UTI in women. The Appraisal of Guidelines for Research and Evaluation (AGREE) II instrument is often used to assess the methodological quality of CPGs in other areas [12]. We therefore aimed to assess the methodological quality of CPGs for recurrent UTI and to summarize recommendations to help clinical decision makers choose the correct CPG for treatment or diagnostics.

## Evidence acquisition

### Study design

We conducted a systematic assessment of the quality of CPGs on recurrent UTI in women using the AGREE II instrument. The protocol for this review was published in PROSPERO under ID CRD42020142882.

### Search strategy and selection of CPGs

Searches of PubMed and EMBASE were performed using defined search terms for UTI and CPG to identify eligible CPGs published between 1 January 2000 and 1 June 2021 (see Supplementary File 1 for full details). The reference lists of all relevant CPGs were also screened manually to identify any CPGs that may have been missed. Finally, two appraisers—J.J.P., a PhD student and recurrent urinary tract infections/urology resident, and M.H.B., a general practitioner/epidemiologist with a special interest in urology and experience in systematic reviews—independently checked the identified literature. Only full CPGs available in English, Dutch, or Spanish that contained recommendations on prevention or diagnostics for recurrent UTI in adult women were included. Disagreements were resolved by discussion and consensus.

### Quality assessment

All CPGs were reviewed by four members from an international multidisciplinary team comprising four urologists, two epidemiologists/methodologists, one urology resident/PhD candidate, and one general practitioner/epidemiologist. CPGs were distributed among the reviewers based on language. We used the AGREE II instrument to appraise the quality of the included CPGs [12]. AGREE II categorizes 23 key items into six domains that each captures a unique dimension of a CPG’s quality: scope and purpose, stakeholder involvement, rigour of development, clarity and presentation, applicability, and editorial independence. This is followed by two global rating items.

Scope and purpose concerns the overall aims of the CPG, the specific health questions, and the target population. Stakeholder involvement focuses on the extent to which the CPG was developed by appropriate stakeholders and represents the views of its intended users. Rigour of development relates to the process for gathering, synthesizing, and updating the evidence, and for formulating recommendations. Clarity and presentation deals with the language, structure, and format of the CPG. Applicability pertains to the barriers and facilitators to implementation, strategies to improve uptake, and resource implications when applying the CPG. Editorial independence is concerned with the formulation of recommendations not being unduly biased by competing interests.

Each item is rated on a 7-point Likert-type scale (1–7, strongly disagree to strongly agree). We performed a calibration review to ensure homogeneity in the assessment among reviewers by having a single CPG assessed by all reviewers. All the scores were compared before the discussion meeting. In this meeting discrepancies between scores were discussed. We concluded that urologists, microbiologists, general practitioners, gynaecologists, and statisticians were relevant professional groups for stakeholder involvement. After reviewers had scored all CPGs, a discussion meeting was organized for any item with a discrepancy of more than three points or where reviewers found different information. This led to individual scores being adjusted before analysis.

### Prevention and diagnostics for recurrent UTI

In addition to assessing methodological quality, we compiled a list of recommended preventive strategies and diagnostics (e.g., urodynamics, ultrasound, or cystoscopy) and rated the strength of those recommendations, which could vary between each CPG based on the framework used. We summarized the CPG definitions by level of evidence and strength of recommendation.

### Data analysis

We performed a descriptive analysis and calculated domain scores by adding the scores of individual domain items and scaling the total as a percentage of the maximum possible score for that domain: [(obtained score) – (minimum possible score)]/[(maximum possible score) – (minimum possible score)]. The minimum possible score was the number of items multiplied by the number of reviewers, and the maximum possible score was the number of items multiplied by the number of reviewers, multiplied by 7 (the highest possible score) [12]. The domain scores are presented per domain per CPG as percentages with the mean score per domain for all CPGs.

### Identifying high-quality CPGs

The appraisers considered the overall quality of the CPGs, rating each as recommended, recommended with modifications, or not recommended [13]. Rigour of development has been considered to have the most direct effect on the quality of a CPG [14]. We classified CPGs as high quality when rigour of development and at least two other domains scored ≥ 60%, as in previous AGREE reviews [6–10].

## Evidence synthesis

### CPG selection and characteristics

**Fig. 1:**
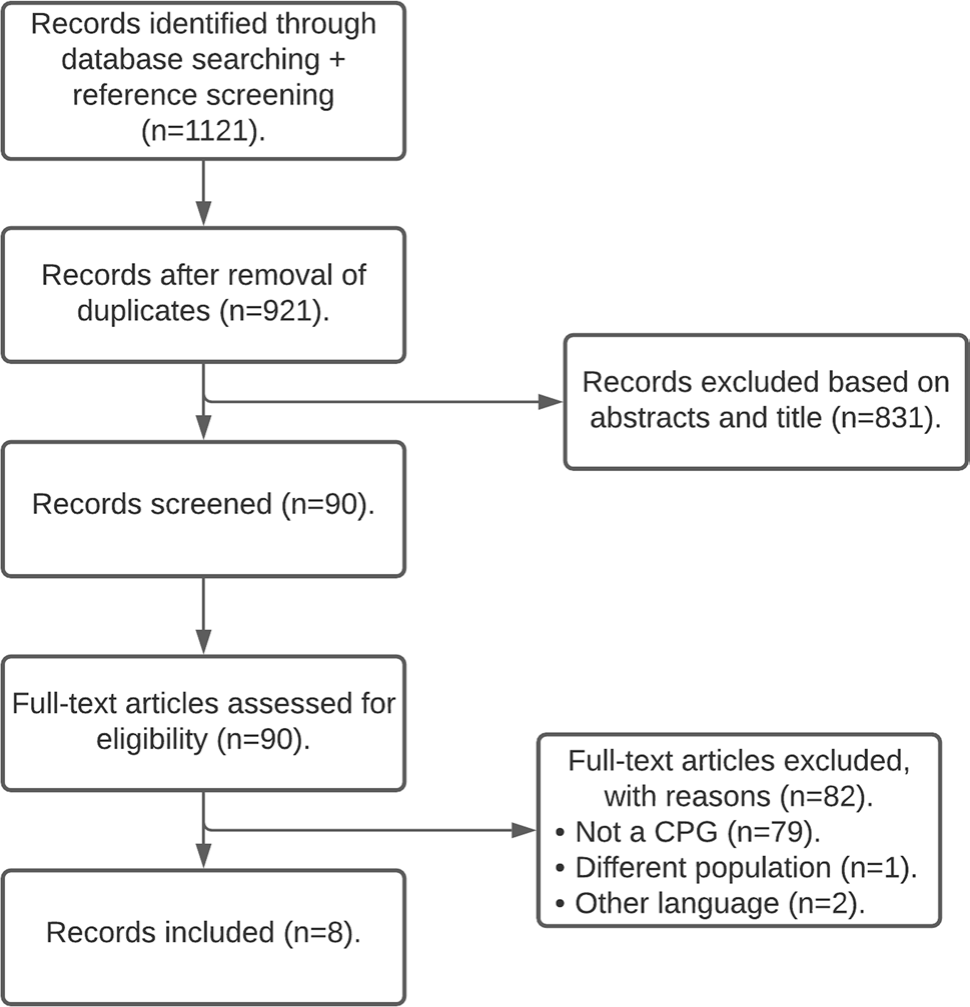
Flow chart of included CPGs

The systematic search revealed 1129 articles, of which 921 remained after removing duplicates and 88 remained after title and abstract screening; ultimately, 8 eligible CPGs were identified by full-text assessment [15–23] (Fig. 1). All CPGs were published between 2013 and 2021, and their characteristics are summarized in Table 1.

**Table 1:** Summary of guideline characteristics

Guideline ref	Year of publication	Responsible agency	Nation	Method used to assess the certainty of evidence	General / recurrent UTI	Population
[16]	2021	EAU	Europe	Modified GRADE	General	Male/female
[15]	2020	NVU	The Netherlands	GRADE	General	Male/female
[22]	2018	AUA/CUA/SUFU	US and Canada	Not specified	Recurrent	Female
[17]	2018	NICE	UK	GRADE	General	Male/female
[20, 21]	2017	AMWF	Germany	OCEBM	General	Male/female
[18]	2017	KAUTII	Korea	Not available	Recurrent	Female
[14]	2014	COMEGO	Mexico	GRADE	Recurrent	Female
[19]	2010	SUA	Spain	OCEBM	Recurrent	Female

Abbreviations: AMWF, German Association of Scientific Medical Societies in Germany; AUA, American Urology Association; COMEGO, Colegio Mexicano de Especialistas en Ginecología y Obstetricia; CUA, Canadian Urology Association; EAU, European Association of Urology; GRADE, Grading of Recommendations Assessment, Development and Evaluation; KAUTII, The Korean Association of Urogenital Tract Infection and Inflammation; NICE, National Institute for Health and Clinical Excellence; NVU, Nederlandse Vereniging voor Urologie; OCEBM, Oxford Centre for Evidence-Based Medicine; SUA, Spanish Urology Association; SUFU, Society of Urodynamics, Female Pelvic Medicine and Urogenital Reconstruction; UTI, urinary tract infection

Two CPGs were written by international (urological) associations [17, 23], with the remainder written by national urological associations of high-income countries [15, 16, 18–22]. Six CPGs [16–22] covered the full scope of UTI with a subsection for recurrent UTI and two CPGs focused on recurrent UTI only [15, 23]. Seven CPGs [16–23] covered both prevention and diagnostics, and one CPG considered only prevention [18].

### CPG appraisal using AGREE II

Table 2 shows the standardized scores per AGREE II domain for the CPG appraisal.

**Table 2:** Standardized scores by clinical practice guideline domain (AGREE II)

Reference	Guideline	Domain						Overall recommendation
		1	2	3	4	5	6	
[16]	EAU	83	54	81	90	23	92	Yes
[15]	NVU	88	81	70	86	68	88	Yes
[22]	AUA/CUA/SUFU	73	38	69	89	7	58	Yes, with modifications
[17]	NICE	51	68	83	74	40	81	Yes, with modifications
[20, 21]	AMWF	71	75	60	72	4	71	Yes, with modifications
[18]	KAUTII	24	3	5	36	4	29	No
[14]	COMEGO	88	46	48	69	7	56	No
[19]	SUA	60	42	39	82	4	33	No

The guidelines are sorted according to Table 1. All scores are presented as %

AGREE II domains are as follows: domain 1 = scope and purpose; domain 2 = stakeholder involvement; domain 3 = rigour of development; domain 4 = clarity of presentation; domain 5 = applicability; domain 6 = editorial independence

Abbreviations: AGREE II, Appraisal of Guidelines, Research, and Evaluation II; AMWF, German Association of Scientific Medical Societies in Germany; AUA, American Urology Association; COMEGO, Colegio Mexicano de Especialistas en Ginecología y Obstetricia; CUA, Canadian Urology Association; EAU, European Association of Urology; GRADE, Grading of Recommendations Assessment, Development and Evaluation; KAUTII, The Korean Association of Urogenital Tract Infection and Inflammation; NICE, National Institute for Health and Clinical Excellence; NVU, Nederlandse Vereniging voor Urologie; OCEBM, Oxford Centre for Evidence-Based Medicine; SUA, Spanish Urology Association; SUFU, Society of Urodynamics, Female Pelvic Medicine and Urogenital Reconstruction; UTI, urinary tract infection

#### Domain 1: Scope and purpose

Reviewers considered six of the CPGs to be of high quality in this domain (range 25%–88%) [15–18, 21–23]. The overall objective was well reported for most CPGs, but scores were low for the description of the specific health questions being covered.

#### Domain 2: Stakeholder involvement

Three CPGs were of high quality in this domain (range 3%–81%). The CPG development groups typically included individuals from all relevant professions. However, efforts were not made to seek the views and preferences of the target population or those efforts were poorly described.

#### Domain 3: Rigour of development

Five CPGs were of high quality in this domain (range 5%–83%) [16–18, 21–23]. Scores varied from low to high across all items for all CPGs. Several classification systems were used to grade the level of evidence (LoE), including the Grading of Recommendations Assessment, Development, and Evaluation (GRADE), the Oxford Centre for Evidence-Based Medicine (OCEBM), and a modified version of GRADE (Table 1). For a description of each classification system see Table 3. The updating procedures for most CPGs were either poorly reported or not reported at all.

**Table 3:** Summary of guideline definitions for level of evidence and strength of recommendation

GRADE level of evidence	
High	We are very confident that the true effect lies close to that of the estimate
Moderate	We are moderately confident in the effect estimate: the true effect is likely to be close to the estimate, but there is a possibility that it is substantially different
Low	Our confidence in the effect estimate is limited: the true effect may be substantially different from the estimate
Very low	We have very little confidence in the effect estimate: the true effect is likely to be substantially different from the estimate
GRADE strength of recommendations	
Strong/weak	Recommendations are characterized as strong or weak (alternative terms, conditional or discretionary) according to the quality of the supporting evidence and the balance between desirable and undesirable consequences of the alternative management options
EAU level of evidence	
1a	Evidence obtained from meta-analysis of randomized trials
1b	Evidence obtained from at least one randomized trial
2a	Evidence obtained from one well-designed controlled study without randomisation
2b	Evidence obtained from at least one other type of well-designed quasi-experimental study
3	Evidence obtained from well-designed non-experimental studies, such as comparative studies, correlation studies, and case reports
4	Evidence obtained from expert committee reports or opinions or clinical experience of respected authorities
EAU strength of recommendations	
Strong/weak	The strength of each recommendation is determined by the balance between desirable and undesirable consequences of alternative management strategies, the quality of the evidence (including certainty of estimates), and the nature and variability of patient values and preferences
OCEBM level of evidence (therapy/prevention)	
1a	Systematic review (with homogeneity) of RCTs
1b	Individual RCT (with narrow confidence interval)
1c	All or none
2a	Systematic review (with homogeneity) of cohort studies
2b	Individual cohort study (including low-quality RCT; e.g., < 80% follow-up)
2c	“Outcomes” research; ecological studies
3a	Systematic review (with homogeneity) of case-control studies
3b	Individual case-control study
4	Case series (and poor-quality cohort and case-control studies)
Oxford strength of recommendations	
Grade A	Consistent level 1 studies
Grade B	Consistent level 2 of 3 studies ***or*** extrapolations from level 1 studies
Grade C	Level 4 studies ***or*** extrapolations from level 2 or 3 studies
Grade D	Level 5 evidence ***or*** troublingly inconsistent or inconclusive studies of any level
AUA/CUA/SUFU level of evidence	
Grade A	Well-conducted and highly generalizable RCT or exceptionally strong observational studies with consistent findings
Grade B	RCTs with some weaknesses of procedure or generalizability or moderately strong observational studies with consistent findings
Grade C	RCTs with serious deficiencies of procedure or generalizability or with extremely small sample sizes, or observational studies that are inconsistent, have small sample sizes, or have other problems that potentially confound data interpretation
Clinical principle	A statement about a component of clinical care that is widely agreed upon by urologists or other clinicians for which there may or may not be evidence in the medical literature
Expert opinion	A statement achieved by Panel consensus and based on members clinical training, experience, knowledge, and judgement for which there is no evidence
AUA/CUA/SUFU strength of recommendations	
Strong	Directive statements that an action should (benefits outweigh risks/burdens) or should not (risks/burdens outweigh benefits) be undertaken because net benefit or net harm is substantial
Moderate	Directive statements that an action should (benefits outweigh risks/burdens) or should not (risks/burdens outweigh benefits) be undertaken because net benefit or net harm is moderate
Conditional	Non-directive statements used when the evidence indicates that there is no apparent net benefit or harm or when the balance between benefits and risk/burden is unclear

Abbreviations: AUA, American Urology Association; CUA, Canadian Urology Association; EAU, European Association of Urology; GRADE, Grading of Recommendations Assessment, Development and Evaluation; OCEBM, Oxford Centre for Evidence-Based Medicine; RCT, randomized controlled trial; SUFU, Society of Urodynamics, Female Pelvic Medicine and Urogenital Reconstruction

#### Domain 4: Clarity of presentation

Seven CPGs were of high quality in this domain (range 36%–90%) [15–18, 20–23]. Most also scored high on all items, with only one scoring low across all items [19].

#### Domain 5: Applicability

One CPG was of high quality (range 4%–68%), having been developed using the AGREE II tool [16]. Otherwise, the CPGs scored low on all items in this domain, though higher scores were achieved for the provision of tools and advice on how to put the recommendations into practice.

#### Domain 6: Editorial independence

Four CPGs were of high quality in this domain (range 29%–92%) [16–18, 21, 22]. The funding agency and potential conflict of interests (COI) were often described in the CPG. However, if and how funding potentially influenced CPG development, as well as how COIs were sought, were poorly reported.

### Overall CPG recommendations

Five CPGs were classified as being of sufficiently high quality to be recommended by the reviewers, scoring > 60% in at least three domains, including rigour of development (Table 2). Overall, two could be recommended outright [16, 17], three could be recommended with modifications [18, 21–23], and three could not be recommended [15, 19, 20].

### Individual recommendations

A summary of all recommendations and the level of evidence is presented in Table 4.

**Table 4:** Summary of recommendations and level of evidence of preventive measures

Preventive therapy	EAU	NVU	AUA/CUA/SUFU	NICE	AMWF	KAUTII	COMEGO	SUA
Behavioural modifications	Weakly recommended	Recommended	Specific recommendations	Recommended	Recommended	Recommended		Recommended
Hormonal replacement	Weakly recommended	Weakly recommended	Moderately recommended	Recommended	Recommended	Recommended	Strongly recommended	
Immunoactive prophylaxis	Strongly recommended		Inconclusive		Recommended	Recommended	Strongly not recommended	Strongly recommended^†^
Probiotics	Inconclusive	Weakly not recommended	Inconclusive	Inconclusive		Recommended	Strongly not recommended	Inconclusive^†^
Cranberry supplements	Inconclusive	Weakly recommended	Conditionally recommended ^††^	Recommended	Inconclusive	Recommended	Strongly recommended	Recommended
D-mannose	Inconclusive	Weakly recommended	Inconclusive	Recommended	Recommended			
Endovesical instillations	Inconclusive	Weakly recommended	Inconclusive					Recommended
Antibiotic prophylaxis	Strongly recommended	Weakly recommended	Moderately recommended	Recommended	Recommended	Recommended	Strongly recommended	Recommended
Methenamine			Inconclusive	Inconclusive		Inconclusive		

^†^The guideline follows the EAU recommendations

^††^Limited use of cranberry juice in diabetic patients due to high sugar content

Abbreviations: AMWF, German Association of Scientific Medical Societies in Germany; AUA, American Urology Association; COMEGO, Colegio Mexicano de Especialistas en Ginecología y Obstetricia; CUA, Canadian Urology Association; EAU, European Association of Urology; GRADE, Grading of Recommendations Assessment, Development and Evaluation; KAUTII, The Korean Association of Urogenital Tract Infection and Inflammation; N.A., not available; NICE, National Institute for Health and Clinical Excellence; NVU, Nederlandse Vereniging voor Urologie; OCEBM, Oxford Centre for Evidence-Based Medicine; SUA, Spanish Urology Association; SUFU, Society of Urodynamics, Female Pelvic Medicine and Urogenital Reconstruction; UTI, urinary tract infection

#### Non-antibiotic prevention

Non-antibiotic prophylaxis in CPGs comprised behavioural modifications, hormonal replacement therapy, immunoactive prophylaxis, probiotics, cranberry supplements, D-mannose, and endovesical instillations.

Six CPGs recommended giving advice on behavioural modifications because such advice is harmless and might benefit some patients [16–22]. The recommended behavioural modifications differed between the CPGs and include: increase water intake, avoid using spermicides and intimate irritants, front to back wiping, post-coital hygiene and using cotton underwear. One did not mention behavioural modifications [15]. Vaginal hormonal replacement was recommended in seven CPGs [15–19, 21–23], and one CPG did not mention this therapy [20]. Immunoactive prophylaxis was recommended in four CPGs [17, 19–22], though one did not recommend it [15], two did not mention it [16, 18], and one did not offer firm advice because of the limited body of evidence [23]. Lactobacillus was not recommended in two CPGs [15, 16] and was recommended in two [19, 21, 22], whereas four deemed the evidence inconclusive [17, 18, 20, 23]. Six CPGs recommend the use of cranberry products [15, 16, 18–20, 23] and two considered the available evidence inconclusive [17, 21, 22]. Three CPGs recommended [16, 18, 21, 22] and one did not give a recommendation [19] on D-mannose, while two considered the data inconclusive [17, 23] and two did not mention it at all [15, 20]. One CPG recommended endovesical instillations with hyaluronic acid in combination with chondroitin sulfate [20], one did not give a recommendation on this therapy [19], and two considered the data inconclusive [17, 23]. The other CPGs did not mention this as an option [15, 16, 18, 21, 22]. Methenamine was mentioned in three guidelines, but all stated that there was insufficient evidence to make a recommendation on it [18, 19, 23].

#### Antibiotic prophylaxis

Antibiotic prophylaxis was advised by all the CPGs when behavioural modifications and non-antibiotic prophylaxis have failed. The most common recommended prophylaxis included nitrofurantoin, trimethoprim, and fosfomycin. Other recommended prophylaxis were cotrimoxazole, ciprofloxacin, norfloxacin, cephalexin, cefaclor, and amoxicillin.

In sexually active women, the first-choice antibiotic prophylaxis was postcoital nitrofurantoin or trimethoprim. The recommended duration of antibiotic prophylaxis ranged between 3 and 12 months, with periodic assessment advised. The NICE guidelines advises reassessment every 6 months, whereas the other guidelines do not further specify periodic assessment [18].

#### Diagnostics

A summary of diagnostic recommendations is presented in Table 5.

**Table 5:** Summary of recommendations and level of evidence on the diagnostic work-up for recurrent UTI

Diagnostics	EAU	NVU	AUA/CUA/SUFU	AMWF	KAUTII	Mexican	SUA
Flowmetry		Recommended			Recommended in specific cases^†^		Specific recommendations^††^
Ultrasound	Weakly recommended in specific cases	Recommended in specific cases	Recommended in specific cases	Recommended	Recommended in specific cases	Strongly recommended in specific cases	Recommended
Cystoscopy	Weakly recommended in specific cases	Recommended in specific cases	Recommended in specific cases	Recommended in specific cases	Recommended in specific cases	Strongly recommended in specific cases	Not recommended

^†^Urodynamics are advised in specific cases according to an algorithm in the guideline. No further information can be found

^††^Urodynamics must be considered when lower urinary tract dysfunction is suspected

Abbreviations: AMWF, German Association of Scientific Medical Societies in Germany; AUA, American Urology Association; COMEGO, Colegio Mexicano de Especialistas en Ginecología y Obstetricia; CUA, Canadian Urology Association; EAU, European Association of Urology; GRADE, Grading of Recommendations Assessment, Development and Evaluation; KAUTII, The Korean Association of Urogenital Tract Infection and Inflammation; NICE, National Institute for Health and Clinical Excellence; NVU, Nederlandse Vereniging voor Urologie; OCEBM, Oxford Centre for Evidence-Based Medicine; SUA, Spanish Urology Association; SUFU, Society of Urodynamics, Female Pelvic Medicine and Urogenital Reconstruction; UTI, urinary tract infection

#### Urodynamics

Three CPGs gave recommendations on when to perform urodynamics [16, 19, 20]. One recommended routine flowmetry based on expert opinion [16], one recommended urodynamics in specific cases based on a flowchart [18], and one recommended urodynamics for suspected lower urinary tract dysfunction based on expert opinion [20].

#### Upper tract imaging

Seven CPGs advised against routine imaging of the upper urinary tract. However, one CPG [21] advised that a single sonography should be performed based on the results of a single-center retrospective study [24]. According to these CPGs, imaging was indicated in atypical case or for patients with persistent haematuria, impaired kidney function tests, or poor response to antibiotic treatment.

#### Cystoscopy

All CPGs unambiguously stated that cystoscopy should not be performed routinely for recurrent UTI, but they differed in the indications that warrant cystoscopy. Clearly gross macroscopic haematuria was considered an indication, but one CPG stated that cystoscopy could be omitted if macroscopic haematuria was only present at the time of an active infection in women aged < 40 years with no risk factors for urothelial cell carcinoma [23]. Most CPGs advised that cystoscopy should be performed in atypical cases or when anatomical abnormalities were suspected. The EAU stated that in these cases cystoscopy should be performed without delay [17]. The other CPGs did not specify a time frame [15, 16, 18–23].

## Discussion

Of the eight CPGs identified for the assessment and treatment of recurrent UTI, our multidisciplinary review team could only recommend two as being of sufficiently high quality for use without adjustment. Another three were also considered to be of high quality, but these could only be recommended with modifications. This illustrates the need to improve CPG development on this topic. In line with previous evaluations, the scope and purpose and clarity of presentation domains had the highest scores, while the stakeholder involvement and applicability domains had the lowest scores [14].

The domains requiring further attention from CPG developers are discussed below.

### Domain 2: Stakeholder involvement

It is important that the CPG development group includes professionals from all relevant groups, clearly defines CPG users, and seeks the views and thoughts of the target population [12]. This could be achieved throughout a discussion meeting with patients, including patients in the development group or as external referents. Ideally, these patients should be trained to perform these tasks. Five CPGs in this review did consider patients’ views during their development, but none reported those views [16–18, 21–23]. Simply providing the patients views and thoughts in a supplement helps with this issue. Further improvement for example could be to add “patient recommended” under the levels of evidence.

### Domain 3: Rigour of development

Developmental rigour, including adequate assessment of the level of evidence of recommendations, probably influences the content of a CPG the most [14]. We found various methods for reporting the evidence levels and grading recommendations among the included CPGs, which had the potential to hinder the user’s ability to compare recommendations. GRADE provides a rigorous and explicit framework for rating the quality of evidence and strength of a recommendation, and its use could help to improve the quality of a CPG. Moreover, it is widely adopted and could be used as a standard for developing CPGs.

Consistent with a previous AGREE review, updating procedures were poorly reported, underlining the need for this to change [14]. There has been little research into the time frame for updating CPGs, but intervals between 1 and 5 years have been suggested [25]. Given that studies regarding prevention and diagnostics for recurrent UTI are far less common than those for studies regarding other pathology (e.g., malignancy), we decided that 5 years is an acceptable time frame. On that basis, six of the eight included CPGs may be considered up to date [16–19, 21–23]. Updating a whole guideline is an intensive and time-consuming process. Topics and recommendations often differ in the terms of the need for updating; therefore, partial updating seems like a logical solution.

### Domain 5: Applicability

Applicability was poor, as in previous appraisals of CPGs, with limited reporting of facilitators and barriers, potential resource implications, and monitoring/auditing criteria. CPG development groups might need to consider development and implementation as separate activities [14]. The process of identifying factors should ideally be done early in CPG development to allow developers to include relevant professionals and develop a realistic implementation plan. Another possibility is to inform users of the need to consider these issues locally when implementing a CPG [14]. The costs of the various preventive strategies for recurrent UTI also vary widely [3]. Failure to consider factors such as facilitators and barriers may hinder CPG implementation. Algorithms or pocket versions could help facilitate CPG use, but only three CPGs in this review included such tools [16, 17, 23]. Having easily identifiable key recommendations could also facilitate CPG use. The importance of increasing the applicability of CPGs was demonstrated in a review of physician adherence to CPGs, which found that as many as 38% considered CPGs inconvenient or too difficult to use [26].

### Domain 6: Editorial independence

Transparency on funding and COIs is important for a CPG to be considered trustworthy. Although only one CPG did not provide funding information [19], it was uncommon for the influence of the funder on CPG development to be reported. Most CPGs provided information about potential COIs, but only two described how these were controlled [17, 18]. These findings are consistent with previous AGREE reviews [6–10]. It would be relatively easy to improve scores in this domain by providing COI forms and including more information about the potential influence of the funding body. The Guidelines International Network Board of Trustees (G-I-N BoT) agreed on nine principles for disclosing interests and managing COIs. These principles should be applied when the guideline development group is composed.

### Strength of recommendations on prevention and diagnostics

Surprisingly, CPGs not only made different recommendations but also provided recommendations of varying strengths. This might reflect differences in both the criteria used to define strength and the evidence available in more recent CPGs. For example, recommendations on the use of D-mannose varied from not being recommended to being recommended, with both positions using data from the same meta-analysis. This has been observed in another AGREE review and might be due to the methods of analysis in order to ensure consensus about the strength of recommendations [6]. By contrast, the recommended CPGs were unanimous when recommending behavioural modifications, hormonal replacement therapy, and antibiotic prophylaxis. Discrepancies in recommendations on probiotics, cranberry supplements, and endovesical installation likely reflected the weak scientific evidence for them. The EAU and AMWF guidelines both recommend immune-active prophylaxis based on the results of three independent meta-analyses [27–29], whereas authors of the AUA/CUA/SUFU found the data insufficient [23].

A diagnostics review published in 2018 concluded that flow rate and post-void residual volume should be measured in all women with recurrent UTI [30]. Only one CPG published since has given a recommendation on urodynamics, but it did not include this earlier review [16]. The review also stated that imaging was unlikely to be of value in the absence of upper tract symptoms. Although most CPGs recommend imaging only in specific cases, CPGs published since 2018 do not include this review [16, 17]. The recommended CPGs unambiguously advised that routine cystoscopy need not be performed, a position that is again supported by data in the earlier review but not cited in CPGs since 2018 [16, 17]. Strict recommendations on when to perform cystoscopy are not offered, except for cases of gross haematuria, which might be because the limited numbers of studies and abnormalities make it difficult to conduct a multivariate analysis.

### Implications for practice and research

According to this review, CPG development for recurrent UTI has been suboptimal to date, with only two CPGs able to be recommended without changes [16, 17]. Another three CPGs [15, 19, 20] could not be recommended at all, but three high-quality CPGs could be recommended if serious flaws are modified [18, 21–23]. These guidelines presented flaws in the applicability [18, 21–23], definition of target users [23], patient views and preferences [23], and description of the covered clinical questions [18]. There are several factors that could improve the quality of CPGs for recurrent UTI.

- Given that recommendations for recurrent UTI include self-management and behavioural modifications, it is important to include patients’ preferences.
- A single method for assessing the quality or level of evidence should be used by all CPG development groups to simplify comparison among the various guidelines and aid interpretation.
- There should be plans to update a CPG regularly. Although any time frame between 1 and 5 years is acceptable, a longer interval may be sufficient for recurrent UTI because new evidence is not published very often.
- Dissemination and integration strategies should be discussed with relevant professionals during development.
- CPG developers should incorporate algorithms and pocket versions and should highlight key recommendation to facilitate use.
- Providing a COI form can improve transparency for potential COIs.
- CPG developers should use frameworks to help plan and draft their guidance. These include the Reporting Items for Practice Guidelines in Healthcare (RIGHT) checklist, which differs from the AGREE II in several ways, and the GIN-McMaster checklist, produced by the Guidelines International Network (GIN) and McMaster University [31]. The RIGHT checklist orders items as the developer and reader would encounter them. It includes important items that were not contained in the AGREE checklist, but that should be reported in a CPG: quality assurance, access, suggestions for further research, and limitations. The GIN-McMaster checklist contains a comprehensive list of topics and items outlining the practical steps to consider when developing CPGs [32].
- The AGREE II instrument could serve as a blueprint for CPG development [12].

### Strengths and limitations

A limitation of our review is that we may not have identified all CPGs because they are often not indexed or are used for in-house purposes only. However, it seems reasonable to assume that the quality of CPGs in this grey literature would be lower than that of indexed and peer-reviewed CPGs, leading to us potentially overestimating the overall quality of CPGs on recurrent UTI in this review. Our review was restricted to CPGs written in English, Dutch, or Spanish, potentially introducing bias by excluding those written in other languages. Despite these limitations, this study benefited from a calibration review to ensure homogeneity of assessment among reviewers. All CPGs were also assessed by four reviewers to provide more reliable conclusions. Moreover, we not only assessed the quality of the CPGs but also summarized the recommendations.

### Conclusion

Few existing CPGs on the topic of recurrent UTI can be recommended without modification, while those that require modification have major limitations in domains such as stakeholder involvement, rigour of development, and applicability. Developers could benefit from using checklists, such as AGREE II, to guide future CPG development.

## Supplementary information

ESM 1 (DOCX 12 kb)

## References

[1] Foxman B, Gillespie B, Koopman J, Zhang L, Palin K, Tallman P (2000). Risk factors for second urinary tract infection among college women. Am J Epidemiol.

[2] Wagenlehner F, Wullt B, Ballarini S, Zingg D, Naber KG. Social and economic burden of recurrent urinary tract infections and quality of life: a patient web-based study (GESPRIT). Expert Rev Pharmacoecon Outcomes Res 2018;18(1):107–117. 10.1080/14737167.2017.1359543 28737469

[3] Gaitonde S, Malik RD, Zimmern PE (2019). Financial burden of recurrent urinary tract infections in women: a time-driven activity-based cost analysis. Urology.

[4] Pat JJ, Steffens MG, Witte LPW, Marcelissen TAT, Blanker MH. Comparison of the diagnostic yield of routine versus indicated flowmetry, ultrasound and cystoscopy in women with recurrent urinary tract infections. Int Urogynecol J 2021. 10.1007/s00192-021-04871-2 PMC9343267 34125244

[5] Field MJ, Lohr KN. Clinical practice guidelines. Directions for a new program. Washington: National Academy Pr 1990. 25144032

[6] Brosseau L, Rahman P, Poitras S, Toupin-April K, Paterson G, Smith C (2014). A systematic critical appraisal of non-pharmacological management of rheumatoid arthritis with appraisal of guidelines for research and Evaluation II. PLoS One.

[7] Poitras S, Avouac J, Rossignol M, Avouac B, Cedraschi C, Nordin M (2007). A critical appraisal of guidelines for the management of knee osteoarthritis using appraisal of guidelines research and Evaluation criteria. Arthritis Res Ther.

[8] Yaman ME, Gudeloglu A, Senturk S, Yaman ND, Tolunay T, Ozturk Y (2015). A critical appraisal of the North American Spine Society guidelines with the appraisal of guidelines for research and Evaluation II instrument. Spine J.

[9] Zhang Z, Guo J, Su G, Li J, Wu H, Xie X (2014). Evaluation of the quality of guidelines for myasthenia gravis with the AGREE II instrument. PLoS One.

[10] Hurdowar A, Graham ID, Bayley M, Harrison M, Wood-Dauphinee S, Bhogal S (2007). Quality of stroke rehabilitation clinical practice guidelines. J Eval Clin Pract.

[11] Naber KG, Bonkat G, Wagenlehner FME (2020). The EAU and AUA/CUA/SUFU guidelines on recurrent urinary tract infections: what is the difference?. Eur Urol.

[12] The AGREE Next Steps Consortium. Appraisal of guidelines for research & evaluation II. 2017; Available at: http://www.agreetrust.org. Accessed 08/02, 2021.

[13] Bauer HW, Rahlfs VW, Lauener PA, Blessmann GS (2002). Prevention of recurrent urinary tract infections with immuno-active E. coli fractions: a meta-analysis of five placebo-controlled double-blind studies. Int J Antimicrob Agents.

[14] Alonso-Coello P, Irfan A, Sola I, Gich I, Delgado-Noguera M, Rigau D (2010). The quality of clinical practice guidelines over the last two decades: a systematic review of guideline appraisal studies. Qual Saf Health Care.

[15] del Pilar Velázquez Sánchez, M, Figueroa Damián R, Hernández D, Romero Nava L. Infección recurrente de vías urinarias. Guía de práctica clínica. 2014; Available at: http://www.comego.org.mx/formatos/Guias/%20GPC2015_3.pdf. Accessed 08/04, 2021.

[16] Nederlandse vereniging voor urologie. Richtlijn Urineweginfecties bij volwassenen. 2020; Available at: https://portal.nvu.nl/WebserviceWordpress/qgws.asmx/nvu_get_document?id=90DDE9FF-0557-EA11-A93F-005056B31E13. Accessed 08/02, 2021.

[17] European Association of Urology. EAU guidelines on urological infections. 2021; Available at: https://uroweb.org/guideline/urological-infections/. Accessed 08/02, 2021.

[18] National Institute for Health and Care Excellence. Urinary tract infection (lower): antimicrobial prescribing. 2018; Available at: www.nice.org.uk/guidance/ng109. Accessed 08/02, 2021. 39937934

[19] Lee S, Choe H, Na Y, Kim K, Kim J, Chung J (2017). 2017 guidelines of the Korean Association of Urogenital Tract Infection and Inflammation: recurrent urinary tract infection. Urogenital Tract Infection.

[20] Prieto L, Esteban M, Salinas J, Adot JM, Arlandis S, Peri L (2015). Consensus document of the Spanish urological association on the management of uncomplicated recurrent urinary tract infections. Actas Urol Esp.

[21] Kranz J, Schmidt S, Lebert C, Schneidewind L, Mandraka F, Kunze M (2018). The 2017 update of the German clinical guideline on epidemiology, diagnostics, therapy, prevention, and Management of Uncomplicated Urinary Tract Infections in adult patients. Part II: therapy and prevention. Urol Int.

[22] Kranz J, Schmidt S, Lebert C, Schneidewind L, Mandraka F, Kunze M (2018). The 2017 update of the German clinical guideline on epidemiology, diagnostics, therapy, prevention, and Management of Uncomplicated Urinary Tract Infections in adult patients: part 1. Urol Int.

[23] Anger J, Lee U, Ackerman AL, Chou R, Chughtai B, Clemens JQ (2019). Recurrent uncomplicated urinary tract infections in women: AUA/CUA/SUFU guideline. J Urol.

[24] Parsons S, Cornish N, Martin B, Evans S. Investigation of uncomplicated recurrent urinary tract infections in women. J Clin Urol 2016.

[25] Vernooij RW, Sanabria AJ, Sola I, Alonso-Coello P, Martinez GL (2014). Guidance for updating clinical practice guidelines: a systematic review of methodological handbooks. Implement Sci.

[26] Cabana MD, Rand CS, Powe NR, Wu AW, Wilson MH, Abboud PA (1999). Why don't physicians follow clinical practice guidelines? A framework for improvement. JAMA.

[27] Neto KA, Castilho L, Oliveira RL (2015). Vacuna oral (OM-89) en la profilaxis de infección urinaria recurrente: una revisión sistemática realista con metaanálisis. Actas Urológicas Españolas.

[28] Naber KG, Cho YH, Matsumoto T, Schaeffer AJ (2009). Immunoactive prophylaxis of recurrent urinary tract infections: a meta-analysis. Int J Antimicrob Agents.

[29] Beerepoot MA, Geerlings SE, van Haarst EP, van Charante NM, ter Riet G (2013). Nonantibiotic prophylaxis for recurrent urinary tract infections: a systematic review and meta-analysis of randomized controlled trials. J Urol.

[30] Santoni N, Ng A, Skews R, Aboumarzouk OM (2018). Recurrent urinary tract infections in women: what is the evidence for investigating with flexible cystoscopy, imaging and Urodynamics?. Urol Int.

[31] Chen Y, Yang K, Marusic A, Qaseem A, Meerpohl JJ, Flottorp S (2017). A reporting tool for practice guidelines in health care: the RIGHT statement. Z Evid Fortbild Qual Gesundhwes.

[32] Mc Master University. GIN-McMaster guideline development checklist. 2014; Available at: https://cebgrade.mcmaster.ca/guidecheck.html. Accessed 08/02, 2021.

