# Supplementary material for: Quality appraisal of clinical guidelines for recurrent urinary tract infections using AGREE II: a systematic review
Source: Int Urogynecol J. 2022 Feb 10;33(5):1059–70. doi: 10.1007/s00192-022-05089-6 (PMC9119892; doi:10.1007/s00192-022-05089-6)
Supplement: Supplementary file 1 — (DOCX 12 kb) [file 192_2022_5089_MOESM1_ESM.docx]

**Supplement 1. Pubmed and Embase search strategies.**

**PubMed search strategy**
((("urinary tract infection"[MeSH Terms] OR ("urinary tract"[All Fields] AND "infection"[All Fields]) OR "urinary tract infection"[All Fields]) AND (("practice guideline"[Publication Type] OR "practice guidelines as topic"[MeSH Terms] OR "practice guideline"[All Fields]) OR ("guideline"[Publication Type] OR "guidelines as topic"[MeSH Terms] OR "guideline"[All Fields]) OR "CPG"[All Fields]))) NOT ((("child"[MeSH Terms] OR "child"[All Fields] OR "children"[All Fields]) OR ("paediatrics"[All Fields] OR "pediatrics"[MeSH Terms] OR "pediatrics"[All Fields]))) AND (("2000/01/01"[PDat] : "2021/06/01"[PDat]) AND Humans[Mesh] AND adult[MeSH]

**EMBASE search strategy**
'urinary tract infection':ab,ti AND 'practice guideline' NOT pediatrics AND [2000-2021]/py
